# GNA15 as a regulator of tumor–immune crosstalk: oncogenic signaling, microenvironmental remodeling, and implications for targeted immunotherapy

**DOI:** 10.3389/fimmu.2026.1955711

**Published:** 2026-09-24

**Authors:** Aarka Undar, Shweta Sunil Nambiar, Mukil Srivasan, Abilash Valsala Gopalakrishnan, Sabina Evan Prince, Wuling Liu, Yaacov Ben-David, Babu Gajendran

**Affiliations:** 1 Department of Bio-Medical Sciences, School of Bio Sciences & Technology, Vellore Institute of Technology, Vellore, India; 2 Department of Biotechnology, School of Bio Sciences & Technology, Vellore Institute of Technology, Vellore, India; 3 State Key Laboratory for Functions and Applications of Medicinal Plants, Guizhou Medical University, Guiyang, China

**Keywords:** GNA15, immune evasion, precision immuno-oncology, targeted effector approaches, tumor microenvironment

## Abstract

GNA15 is a Gαq-family G protein whose physiological expression is largely restricted to hematopoietic cells. Nevertheless, aberrant upregulation has been reported in six histologically distinct malignancies, with elevated expression consistently associated with adverse clinical outcomes. Unlike the ubiquitously expressed Gαq and Gα11 paralogs, GNA15 exhibits an expanded receptor-coupling repertoire that extends beyond their canonical GPCR interactions. Despite the diversity of tumor types, its oncogenic functions consistently converge on four major signaling programs: MAPK activation, BTK-dependent signaling, AMPK-mediated metabolic reprogramming, and EMT-associated cellular plasticity. Each of these mechanisms has been functionally validated in at least one cancer model. Across multiple malignancies, elevated GNA15 expression is associated with increased M2 macrophage infiltration, immunosuppressive transcriptional signatures, and poor patient outcomes. These associations remain correlative, however, as no study has yet demonstrated that GNA15 signaling directly drives these immune-related phenotypes. Nevertheless, existing evidence suggests that GNA15-associated changes in the tumor microenvironment could influence the effectiveness of immune checkpoint blockade and may also bear on the success of newer approaches such as engineered cellular therapies, bispecific antibodies, and antibody–drug conjugates. By contrast, the evidence supporting its oncogenic function is considerably more robust: genetic silencing of GNA15 consistently attenuates malignant phenotypes across four independent tumor models, providing the strongest evidence for its therapeutic relevance. Its proposed immunological role, by comparison, remains hypothesis-generating, as it is derived exclusively from correlative observations and has not been evaluated in immune-competent experimental systems. Overall, GNA15 represents a biologically plausible yet clinically unvalidated link between GPCR signaling and tumor–immune regulation. At present, the available evidence supports its development as a prognostic biomarker; whether it also has predictive value for immunotherapy response remains untested and requires prospective validation.

## 1 Introduction

G protein-coupled receptors (GPCRs) form the largest family of cell-surface signaling proteins in humans, comprising more than 800 members. Each receptor converts extracellular cues into intracellular signals through heterotrimeric G proteins built from Gα, Gβ, and Gγ subunits (1). Receptor activation exchanges GDP for GTP on the Gα subunit and releases the Gα subunit from the Gβγ dimer. Each Gα class then couples to a characteristic effector: Gαs stimulates adenylyl cyclase, Gαi/o inhibits it, Gαq/11 activates phospholipase Cβ (PLCβ), and Gα12/13 engages Rho guanine nucleotide exchange factors (1, 2). Dysregulation of this machinery recurs as an oncogenic mechanism across tumor types. GNAS mutations drive pancreatic intraductal papillary mucinous neoplasms, GNAQ/GNA11 mutations account for more than 80% of uveal melanomas through constitutive PLCβ and YAP activation (3), and gain-of-function RHOA mutations are enriched in diffuse-type gastric carcinoma and angioimmunoblastic T-cell lymphoma (4). GPCRs also act directly on the tumor immune compartment. Gα-coupled signaling drives CD8+ T-cell dysfunction through a PKA-dependent axis (5), and GPCR-driven chemokine gradients govern immune-cell recruitment and polarization within tumors (6, 7). These dual roles, as oncogenic drivers and as modulators of anti-tumor immunity, position GPCR-coupled G proteins as candidate determinants of response to targeted immunotherapy. The gene sits on chromosome 19p13.3 and encodes the 374-amino-acid Gα15 protein (8). It was originally described as a hematopoietically restricted transducer with promiscuous coupling to receptors that do not normally engage Gαq-family members, unlike the ubiquitously expressed Gαq and Gα11 (8). Aberrant GNA15 expression has been reported in six malignancies: pancreatic ductal adenocarcinoma (PDAC) (8), ovarian cancer (9–11), thyroid carcinoma (12), acute myeloid leukemia (AML) (13), triple-negative breast cancer (TNBC) (14), and B-cell acute lymphoblastic leukemia (B-ALL) (15), and consistently correlates with adverse clinicopathological features and shorter survival. No single conserved mechanism accounts for this pattern. GNA15 instead draws on a small repertoire of signaling programs: MAPK amplification, BTK coupling, AMPK-driven metabolic reprogramming, and EMT-linked plasticity, that recombine differently across tumor lineages (8, 12–15). The same tumors in which GNA15 is overexpressed also show recurring immune features, including M2-polarized macrophage infiltration in ovarian cancer (9); inflammatory and EMT transcriptional programs at single-cell resolution in TNBC (14); and NF-κB/STAT3 activation across tumor types (8, 14). The recurrence of these findings suggests that GNA15 is more than an incidental oncogenic event, supporting its role as both a driver of tumor growth and a potential contributor to immune evasion. However, it remains unknown whether GNA15 signaling directly influences responsiveness to immunotherapy or whether its coordinated regulation of multiple signaling pathways actively remodels the tumor immune microenvironment. Resolving these questions will require careful discrimination between experimentally validated mechanisms and observations that remain correlative or hypothetical across different tumor types. Such evidence is essential for determining which proposed immune-related functions of GNA15 are sufficiently substantiated for therapeutic translation and which require further experimental investigation.

## 2 Organization of GNA15 signaling and its immune-related effector outputs

### 2.1 Gα15 structural organization and signal transduction

Gα15 maintains the conserved GTPase and helical-domain organization shared by heterotrimeric Gα proteins, including the Switch I–III motifs that coordinate effector interactions during GDP–GTP exchange (1). Its major structural differences relative to Gαq and Gα11 are concentrated within the C-terminal α5 helix and the α4–β6 loop, which constitute the principal receptor-binding interface and underlie its expanded receptor-coupling capacity (1, 8, 16, 17). Following activation, Gα15 promotes PLCβ-dependent cleavage of PIP2 into the second messengers DAG and IP3. These molecules activate complementary signaling cascades: IP3 stimulates Ca²^+^ release from the endoplasmic reticulum, leading to activation of the CaMKK2–AMPK pathway (18), whereas DAG activates PKC, thereby driving MAPK signaling and NF-κB activation through IKK (8). The signaling network is further amplified through non-canonical pathways involving Src-family kinases, PI3K, and STAT3 (8). Together, these interconnected pathways regulate MAPK, NF-κB, STAT3, and PI3K/AKT signaling, which are well recognized for controlling inflammatory cytokine production, macrophage polarization, and immune checkpoint regulation in other tumor contexts. This signaling architecture provides a mechanistic basis for the four recurrent oncogenic programs associated with GNA15-high tumors. Whether it also directly mediates immune modulation, however, has not been demonstrated and is treated as a distinct question. In contrast, Gαs-coupled GPCR signaling suppresses T-cell activation through a PKA-dependent mechanism acting intrinsically within lymphocytes (5). Because Gα15 is predominantly expressed in tumor and myeloid compartments rather than in T cells, any immunological effects are more likely to be mediated indirectly through paracrine signals originating from tumor or stromal cells than through cell-autonomous suppression of lymphocytes. Consistent with this view, a comprehensive single-cell analysis across 19 cancer types linked GPCR-dependent T-cell exhaustion to the Gαs–PKA axis rather than to Gα15 signaling (5). These findings emphasize the need to investigate GNA15-specific paracrine mechanisms of immune regulation rather than to infer T-cell-intrinsic functions from the established Gαs–PKA pathway.

### 2.2 GNA15 versus Gαq and Gα11: structural and functional divergence

GNA15 belongs to the Gq family of heterotrimeric G-protein α-subunits, which in mammals also includes GNAQ, GNA11, and GNA14. Despite this shared ancestry, family members differ sharply in tissue distribution. Gαq and Gα11 are expressed ubiquitously across tissues, whereas Gα15 (the mouse ortholog; its human counterpart is historically designated Gα16) is expressed almost exclusively within the hematopoietic compartment, predominantly in myeloid and B-lineage cells, with more limited representation in other blood cell lineages (8, 16). Gα14 occupies an intermediate pattern, with expression largely restricted to spleen, lung, kidney, testis, and selected hematopoietic progenitors (16). This restricted physiological distribution is central to the oncogenic significance of GNA15: its aberrant appearance in solid epithelial tumors such as PDAC, thyroid carcinoma, and TNBC represents ectopic reactivation of a lineage-restricted gene, rather than dysregulation of a normally ubiquitous signaling node. This distinction carries direct implications for on-target toxicity in any GNA15-directed therapeutic strategy and is revisited. The most consequential functional distinction between GNA15 and its paralogs is the breadth of receptor coupling. Gαq and Gα11 couple selectively to a defined repertoire of Gq-linked GPCRs. Gα15/16, in contrast, couples productively to a substantially broader range of receptors, including many that physiologically signal through Gi- or Gs-coupled pathways, redirecting their output toward PLCβ activation and Ca²^+^ mobilization (16, 17); this property has been exploited pharmacologically for decades as a tool for deorphanizing GPCRs of unknown coupling in heterologous expression systems. This promiscuity is not a generic feature of the Gq family. Still, it has been mapped, through systematic mutagenesis and receptor-coupling assays, to specific structural determinants of Gα15/16: principally the C-terminal α5 helix, which forms the majority of the direct interface with the receptor’s intracellular cavity, together with contributions from the adjacent α4–β6 and β2–β3 loops (17). A defined cluster of divergent residues within the α5 helix broadens the receptor-coupling profile when substituted with sequence from other Gα subfamilies. The reciprocal substitution narrows coupling back toward the canonical Gq-linked repertoire (17), indicating that this region is both necessary and, in combination with the adjacent loops, largely sufficient to explain the promiscuity phenotype at the level of cell-based coupling assays. This structural interpretation rests on site-directed mutagenesis and chimeric-receptor coupling data rather than on a solved atomic structure: at the time of writing, structural (crystallographic or cryo-EM) data directly visualizing a Gα15/16–GPCR complex are markedly more limited than the extensive structural literature available for Gαq-, Gαs-, and Gαi-coupled complexes. This gap is explicitly noted here rather than assumed away, and it is listed as a priority for future structural work. GNA15’s route to oncogenic relevance also differs qualitatively from that of its paralogs. GNAQ and GNA11 are activated in cancer predominantly through recurrent, mutually exclusive hotspot mutations (most commonly at Gln209 or Arg183) that abolish intrinsic GTPase activity and lock the protein in a constitutively active, GTP-bound state; these mutations account for more than 80% of uveal melanomas and drive constitutive PLCβ and YAP activation largely independent of upstream receptor input (3). No comparable recurrent activating mutation in GNA15 has been reported in any of the malignancies reviewed here. Instead, the oncogenic mechanism described across PDAC, AML, thyroid carcinoma, TNBC, ovarian cancer, and B-ALL is transcriptional and, in PDAC, epigenetic: aberrant re-expression of structurally normal, wild-type GNA15 outside its physiological hematopoietic context, rather than a gain-of-function point mutation within it (8, 12–15). This distinction matters for drug discovery. GNAQ/GNA11-mutant disease is, in principle, amenable to mutant-selective or conformation-selective inhibitor strategies analogous to those developed for other constitutively active oncoproteins, whereas GNA15-driven disease instead requires strategies that suppress the expression, delivery, or downstream signaling of an otherwise structurally normal protein. The pharmacological targeting landscape further separates GNA15 from Gαq and Gα11. The macrocyclic depsipeptides YM-254890 and FR900359 are selective, high-affinity inhibitors of Gq-family signaling that have become standard tool compounds for interrogating Gαq/11/14 biology and have shown efficacy in preclinical disease models; both compounds, however, spare Gα15/16, which is evolutionarily more distant within the Gq family and lacks the residues that constitute the YM-254890/FR900359-binding pocket (19). Structure-guided protein engineering has restored partial sensitivity to Gα16 by transplanting the Gαq-type binding pocket, demonstrating that this resistance is a defined, mutable structural property rather than an intrinsic barrier to inhibitor design (19). However, this remains an engineered research tool rather than a therapeutic candidate. Consequently, no selective small-molecule inhibitor of Gα15 currently exists for research or therapeutic use, in contrast to the mature chemical-probe toolkit available for Gαq/11. Taken together, these differences indicate that GNA15 poses a therapeutic problem that is structurally and pharmacologically distinct from the one posed by its Gαq/Gα11 paralogs, rather than a variation on the same problem (**Table 1**). Its restricted physiological expression favors tumor-selective targeting strategies over systemic Gα15 inhibition, given the risk of on-target toxicity to normal hematopoiesis. Its receptor-coupling promiscuity means that the specific GPCR(s) driving pathological Gα15 activation in any given tumor are not yet defined, unlike the comparatively well-characterized receptor inputs to Gαq/11-driven disease. Its activation by aberrant expression, rather than by an activating mutation, removes mutant-selective inhibition as a strategy. The absence of a selective chemical probe leaves genetic silencing and downstream-effector inhibition as the only experimentally validated intervention points at present. These distinctions motivate treating GNA15 as a biologically and therapeutically distinct target rather than extrapolating findings directly from the substantially larger GNAQ/GNA11 literature in uveal melanoma.

**Table 1:** Comparative biology of GNA15/Gα15 versus GNAQ/Gαq and GNA11/Gα11.

Feature	Gα15/GNA15	Gαq and Gα11/GNAQ, GNA11	Evidence basis
Tissue/cellular expression	Hematopoietic-restricted (predominantly myeloid and B-lineage); ectopically re-expressed in several solid tumors	Ubiquitous across tissues	(8, 16)
Receptor-coupling spectrum	Broad/promiscuous; couples productively to many Gi- and Gs-linked receptors in addition to canonical Gq-linked GPCRs	Canonical Gq-linked GPCRs only	(16, 17)
Structural basis of coupling specificity	α5 helix and α4–β6/β2–β3 loops implicated by mutagenesis and receptor-coupling assays; no solved Gα15/16–GPCR structure reported to date	Coupling specificity for canonical receptors is better characterized structurally, drawing on the broader Gq/GPCR structural literature	(1, 17)
Cancer-associated activation mechanism	Transcriptional/epigenetic overexpression of structurally normal, wild-type protein; no recurrent activating mutation reported	Recurrent hotspot activating mutations (Gln209, Arg183) abolishing GTPase activity	(3, 8, 12–15)
Tumor types with direct functional evidence	PDAC, AML, thyroid carcinoma, TNBC, ovarian cancer, B-ALL (genetic silencing studies)	Uveal melanoma (>80% of cases); blue nevi	(3, 8, 9, 12–15)
TME immune regulation	Correlative evidence only (M2 macrophage infiltration, immunosuppressive signatures); no direct causal data	Described in the separate uveal melanoma immunology literature; outside the scope of this review	(3, 9, 14)
Selective pharmacological inhibitors	No available; resistant to YM-254890 and FR900359	YM-254890, FR900359 (high-affinity, selective tool compounds)	(19)
Therapeutic implication	Genetic silencing and downstream-effector inhibition (MAPK, BTK, AMPK/CPT1) are the only experimentally validated intervention points	Direct Gα inhibition and mutant-selective targeting strategies are feasible in principle	Section 5.1

## 3 Recurring oncogenic signaling themes across GNA15-high malignancies

### 3.1 MAPK pathway amplification

Adult AML carries the worst prognosis of any leukemia, with five-year overall survival hovering near 24% (20–22), and it is also the cancer type where the mechanistic case for GNA15-driven MAPK signaling is most complete. GNA15 transcripts are elevated in AML blasts relative to healthy donor PBMCs and further enriched in leukemia stem cells, a pattern built from ImmuCo, ImmuSort, ONCOMINE, and GSE12417 data and cross-validated in 154 newly diagnosed patients against 26 controls (13). High expression independently predicted shorter overall and relapse-free survival after adjusting for age, cytogenetic risk, and FLT3-ITD status (13). Knockdown in Kasumi-1 and SKNO-1 cells reduced colony formation and triggered apoptosis. This was evidenced by increased levels of cleaved caspase-3, PARP, and p53, and decreased levels of Cyclin D1, with increased p27 levels. GNA15 depletion selectively reduced phosphorylation of p38 MAPK, MAPKAPK2 (MK2), and CREB without affecting ERK/JNK activation. Partial restoration of signaling by asiatic acid confirmed that the GNA15–p38–MK2–CREB axis is the principal functional pathway (13). Consistent with these findings, knockdown of GNA15 notably reduced tumor growth in a xenograft model, providing the most compelling *in vivo* evidence to date of its oncogenic function across the tumor types studied. Activation of downstream CREB targets enhances transcription of BCL2, BCL-XL, MCL1, and Cyclin D1, providing a mechanistic explanation for the apoptosis resistance observed in GNA15-high AML cells (13). In papillary thyroid carcinoma (PTC), GNA15 activates a broader MAPK signaling program than that observed in AML. Although PTC is predominantly driven by activating BRAFV600E or RAS mutations that constitutively stimulate MAPK signaling (23), ectopic GNA15 expression further increased cell viability, migration, and invasion, while simultaneously inducing phosphorylation of ERK, JNK, and p38. Conversely, shRNA-mediated silencing of GNA15 abolished these effects (12). Comparison of these tumor models highlights a major principle- whereas Gα15 preferentially signals through p38 in myeloid leukemia, it activates ERK, JNK, and p38 concurrently in thyroid carcinoma. These observations indicate that the prevailing MAPK output is dictated primarily by the lineage-specific repertoire of adaptor proteins rather than by intrinsic properties of Gα15 itself, with important implications for selecting optimal combination therapies. Integrated single-cell RNA sequencing and bulk multi-omics analyses in TNBC, supporting this context-dependent model, identified a distinct malignant cell population (S2) characterized by elevated expression of GNA15, enrichment of multiple oncogenic pathways, and poor clinical outcome (14, 24). Functional silencing further confirmed that GNA15 promotes proliferation, migration, and invasion within this defined TNBC subpopulation. Because these observations were derived from single-cell transcriptomic analyses rather than bulk profiling, they suggest that high GNA15 expression marks a specific malignant cellular state instead of reflecting generalized transcriptional changes. Independently, p38 MAPK and NF-κB signaling downstream of Gα15 have been implicated in regulating both pro-inflammatory (IL-6, IL-8, TNF-α) and immunosuppressive (IL-10, TGF-β) cytokine production in other tumor settings (25, 26). However, this cytokine-regulatory function has not yet been investigated for GNA15 in AML, TNBC, or thyroid carcinoma. The established GNA15 knockdown models in these malignancies provide an ideal experimental platform for cytokine profiling and tumor–immune co-culture studies to evaluate this possibility directly.

### 3.2 BTK-coupled signaling as an upstream amplifier

In thyroid carcinoma, silencing Bruton’s tyrosine kinase (BTK) alongside GNA15 substantially blunted both tumorigenesis and MAPK phosphorylation, while GNA15 overexpression partially compensated for the loss of BTK in maintaining MAPK activity (12). These findings represent direct functional evidence rather than a mechanistic inference. They position Gα15 as an upstream signaling hub capable of concurrently activating both BTK-dependent and BTK-independent MAPK pathways, an observation of particular significance because it occurs outside the canonical B-cell receptor signaling context in which BTK is traditionally characterized (12, 27, 28). This expands the role of BTK to include non-canonical signaling functions in an epithelial solid tumor. In B-cell malignancies, BTK-driven NF-κB activation promotes PD-L1 transcription (27, 28). Accordingly, a GNA15-mediated, BTK-independent route to NF-κB activation could potentially maintain checkpoint molecule expression despite BTK inhibition in GNA15-high thyroid tumors. Although this proposed resistance mechanism remains untested, it is mechanistically supported by the signaling framework established in thyroid carcinoma. It can be evaluated using the same experimental models that defined the GNA15–BTK axis. Should GNA15 overexpression compensate for BTK inhibition, persistent GNA15-dependent NF-κB signaling may diminish the immunological benefit of BTK-targeted therapy in this specific tumor setting (**Figure 1**).

**Figure 1:**
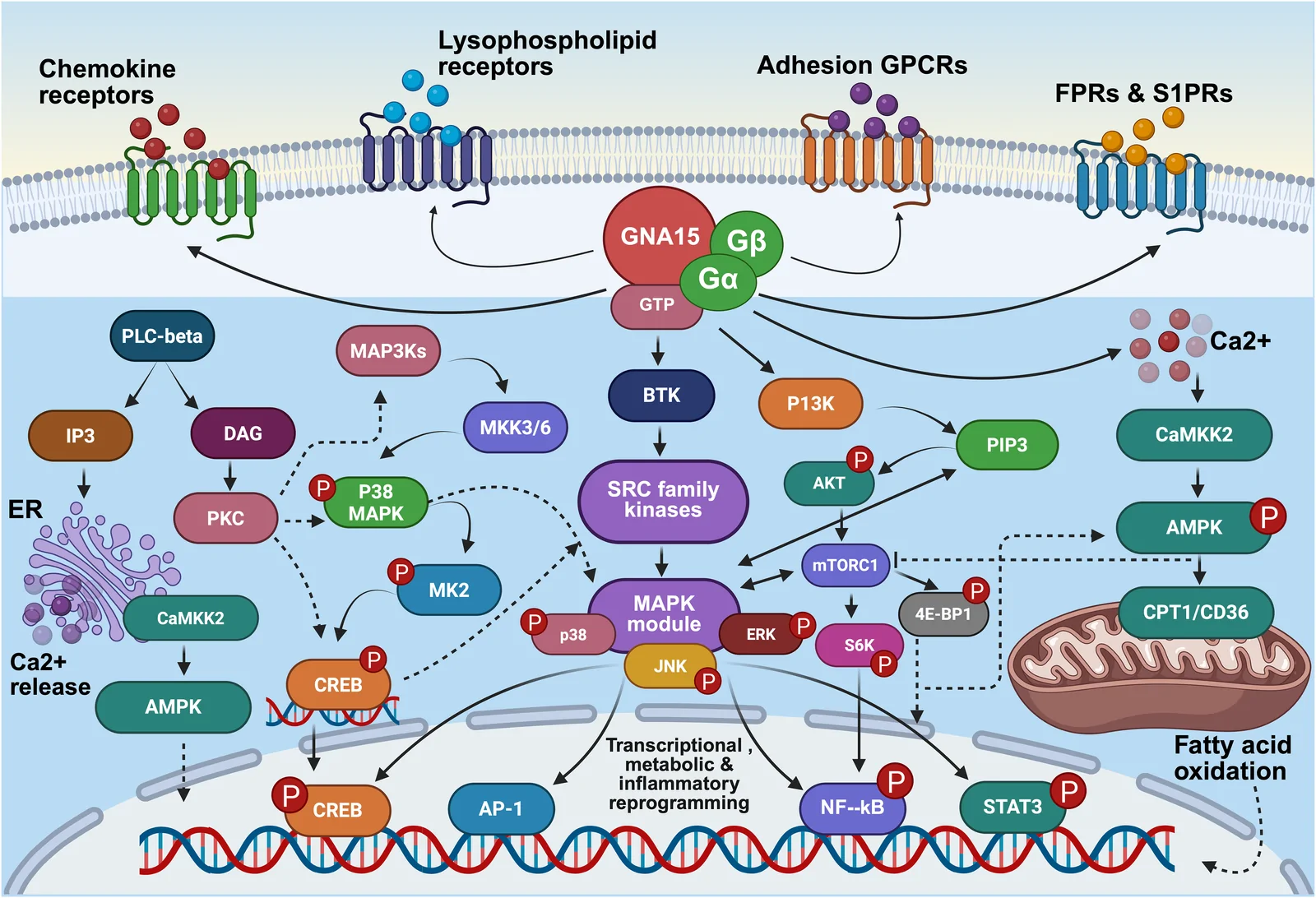
Integrated oncogenic signaling organization of GNA15 in human malignancies. GNA15 functions as a signal-transduction node linking aberrant GPCR activity to multiple oncogenic programs. On activation, GAlpha15-coupled PLCBeta hydrolyzes PIP2 into IP3 and DAG, driving intracellular Ca²^+^ mobilization and PKC activation. Downstream signaling converges on the p38–MK2–CREB axis, BTK-associated MAPK cascades, NF-KappaB, PI3K–AKT–mTOR, and STAT 3, and engages AMPK-driven fatty-acid oxidation. These signaling pathways collectively regulate transcriptional reprogramming, survival signaling, proliferation, invasion, metastatic dissemination, metabolic adaptation, and therapeutic resistance, illustrating how canonical and non-canonical GNA15-dependent pathways converge on a shared oncogenic output across tumor types. (Arrows indicate direction of signaling. →, direct activating step supported by established evidence; ⇢ (dashed), indirect, secondary, or mechanistically inferred connection; ↔, bidirectional/reciprocal regulation; ⊣, inhibitory interaction; ↔, phosphorylated/active form of the indicated protein).

### 3.3 AMPK-driven metabolic rewiring

Among GNA15-high malignancies, B-ALL provides the clearest example of GNA15-driven metabolic reprogramming, where GNA15 promotes drug resistance through AMPK-orchestrated fatty acid oxidation (FAO). Overexpression raised AMPK Thr172 phosphorylation, elevated the FAO effectors CPT1 and CPT2, and increased surface CD36. Untargeted metabolomics confirmed enrichment of the AMPK pathway and glycerophospholipid rewiring (15). Two inputs converge to sustain this activation of IP3-driven Ca²^+^ release through CaMKK2 (18), and DAG-activated PKC acting through LKB1 (29). AMPK then relieves ACC1/ACC inhibition of CPT1, increasing mitochondrial fatty acid import and Beta-oxidation flux to supply ATP and NADPH under genotoxic stress (30). Pharmacological AMPK inhibition with dorsomorphin reversed the program, and CPT1 inhibition with etomoxir independently reversed chemoresistance. Altogether, these findings establish the GNA15–AMPK–FAO–chemoresistance axis as one of the few GNA15-associated mechanisms with genuine pharmacological, not just genetic, validation. A distinct, epigenetically driven form of lineage reprogramming occurs in PDAC, where promoter demethylation derepresses GNA15, reactivating a hematopoietically restricted gene within an epithelial lineage (8). This is a different upstream mechanism from the AMPK-FAO axis described in B-ALL, though both illustrate how GNA15 dysregulation can arise through distinct routes in different tissue contexts. In PDAC, high expression levels correlate with poor prognosis, and CRISPR/Cas9 or shRNA-mediated silencing reduces tumor cell motility and invasiveness (8). These findings position GNA15 within a broader spectrum of lineage-plasticity programs through which solid tumors reacquire hematopoietic-like transcriptional features. In other malignancies, comparable reprogramming events have been associated with therapeutic resistance and altered immune surveillance, although such relationships have not yet been demonstrated in GNA15-driven PDAC. Likewise, it remains unknown whether the AMPK–FAO pathway described in B-ALL is also active in PDAC or whether its activation could limit fatty acid availability for tumor-infiltrating T cells. These possibilities remain important but untested hypotheses rather than established components of PDAC biology.

### 3.4 EMT and cellular plasticity

Three effectors already implicated in GNA15 signaling, PKC, ERK2, and STAT3, are each independently known to stabilize mesenchymal transcription factors: PKC stabilizes Snail and ZEB1, ERK2 stabilizes ZEB1 and Slug, and STAT3 drives transcription of Vimentin and Fibronectin (8, 14). To date, no study has directly demonstrated that GNA15 activation coordinates these downstream effectors into an integrated EMT program in any cancer type. The strongest supporting evidence comes from the TNBC S2 cluster described above, which represents a discrete malignant cell population rather than generalized transcriptional heterogeneity (14). However, it remains unknown whether this cluster exhibits EMT characteristics, reduced MHC-I expression, or altered susceptibility to NK-cell-mediated cytotoxicity. Accordingly, these features should be regarded as testable hypotheses in TNBC rather than established properties of GNA15-driven biology. Prognostic association and functional validation across these tumor types represent two distinct types of evidence and should not be confused. Regarding prognosis: high GNA15 expression independently predicts shorter overall and relapse-free survival in cytogenetically normal AML (13), it is linked to poorer outcomes in PDAC (8) and thyroid carcinoma (12), it enhances prediction beyond a serous ovarian cancer programmed cell death (PCD)-related gene signature that associates high expression with T- and B-cell receptor signaling and low expression of ribosomal biogenesis (10) and it identifies a more aggressive subpopulation in TNBC (14). These are correlative, cancer-specific associations rather than evidence of a shared mechanism. Functional validation is more consistent, however, shRNA or CRISPR/Cas9 silencing of GNA15 has reproducibly reversed tumorigenic phenotypes in PDAC (8), AML (13), ovarian cancer (9), and TNBC (14), which is as close to direct causal validation of GNA15 as a therapeutic target as the current evidence gets in any of these tumor types. This integration across otherwise unrelated malignancies argues that GNA15 overexpression recurrently confers a growth or survival advantage, even though the specific downstream program achieving that advantage MAPK isoform selection, BTK coupling, AMPK-FAO rewiring, or EMT-associated plasticity differs by tissue context. Whether tumor microenvironment remodeling is a shared consequence of this recurrent oncogenic role, or an unrelated, tumor-specific correlate, remains an open question.

## 4 GNA15 and tumor immune microenvironment remodeling

### 4.1 Myeloid reprogramming and innate immune remodeling

In ovarian cancer, GNA15 emerged as a hub gene positively correlated with M2 macrophage infiltration scores and the markers CD163 and CD206, as determined by integrative TCGA transcriptomic analysis, validated by immunohistochemistry and qRT-PCR in clinical specimens (9). This is a correlation rather than a demonstrated mechanism. CRISPR/Cas9- and shRNA-mediated GNA15 knockdown in OVCAR5 cells independently reduced cell proliferation, confirming a tumor-cell-intrinsic oncogenic role distinct from the TAM association, which remains correlative (9). Several mechanistic routes could link GNA15 to M2 polarization of secretion of M2-polarizing cytokines (IL-10, IL-6, CCL2, CSF-1) via PKC–NF-κB or STAT3 activation, altered exosomal cargo, and GPCR-mediated chemokine gradients that recruit monocytes and direct their differentiation (6, 9). These mechanisms are not mutually exclusive, and which one predominates likely differs by tumor type. Resolving that question will require spatial rather than bulk transcriptomic data. A useful precedent exists in colorectal liver metastasis, where M2-like macrophages occupy spatial niches distinct from cytotoxic T cells (31). A comparable spatial analysis of GNA15-high ovarian cancer would show whether GNA15-expressing tumor cells, M2-polarized TAM niches, and functional T cells occupy overlapping or adjacent territories, rather than being averaged together by bulk sequencing, which cannot resolve their spatial organization. In AML, GNA15-high cells activate NF-κB and produce IL-6, IL-8, and TNF-α (13, 25). These cytokines would be expected to polarize bone marrow-resident macrophages and myeloid progenitors toward immunosuppressive phenotypes, though whether this cytokine output actually shields leukemia stem cells from immune surveillance *in vivo* has not been examined. The AML and ovarian cancer findings altogether suggest that GNA15-driven myeloid reprogramming is not restricted to a single lineage, a pattern that could be tested by immune deconvolution of existing transcriptomic datasets before undertaking functional studies in each tumor type individually. Myeloid-derived suppressor cells (MDSCs) represent an unexplored extension of this idea. STAT3, activated downstream of GNA15 through both canonical and non-canonical routes (8), is an established driver of MDSC expansion elsewhere in oncology, so a parallel role in GNA15-high disease is plausible. No published dataset, however, has directly measured MDSC frequency in GNA15-stratified specimens.

### 4.2 Cytokine and chemokine signaling circuits

NF-κB and STAT3, both activatable downstream of Gα15, are the principal transcriptional drivers of the cytokine output described. NF-κB induces IL-6, IL-8, TNF-α, and CCL2, while STAT3 induces IL-10 and VEGF and impairs dendritic cell maturation (8). Gastric cancer offers a template for how such a circuit could become self-reinforcing, as TAM-derived TNF-α and IL-6 activate tumor-cell NF-κB/STAT3 to induce PD-L1 expression, creating a macrophage–tumor loop that sustains both immune evasion and proliferation (32). A comparable circuit is plausible in GNA15-driven disease, in which tumor-derived IL-6 sustaining macrophage STAT3 activity, IL-10 output, and further M2 polarization would be expected to limit checkpoint inhibitor efficacy unless the upstream GNA15 signal were co-targeted. This remains a hypothesis rather than a demonstrated mechanism, and testing it will require immune-competent co-culture and *in vivo* systems rather than further correlative analysis.

### 4.3 Metabolic immune regulation

The GNA15–AMPK–FAO axis established in B-ALL raises a specific question, Does the same metabolic program that sustains tumor cell survival also deplete tumor-infiltrating T cells of fatty acid substrate? This is conceptually distinct from, though potentially additive to, the cytokine-based immunosuppression described above. FAO-dependent tumor programs are generally linked to nutrient competition with infiltrating lymphocytes elsewhere in the literature, and CD36-mediated fatty acid uptake is associated with reduced fatty acid availability for T-cell effector differentiation across several tumor models (30). A metabolically restrictive TME driven specifically by GNA15-orchestrated CD36 upregulation, however, has no direct experimental support in GNA15-high tumors. Another potential mechanism is suggested by findings in colorectal cancer, where CD36-dependent lipid uptake promotes M2 macrophage polarization (33). Similarly, higher CD36 expression in GNA15-high tumor cells may promote lipid transfer to nearby tumor-associated macrophages, suggesting a form of immune regulation distinct from direct metabolic competition with T cells. This possibility has not yet been investigated. Importantly, the GNA15–AMPK–CPT1/2–FAO signaling axis has already been pharmacologically validated in B-ALL using etomoxir and dorsomorphin (15). The unresolved question is therefore not whether this pathway exists but rather its immunological impact. Metabolic flux analyses in co-culture systems comprising GNA15-high tumor cells and T cells would provide a direct means to distinguish substrate competition from paracrine mechanisms of immune suppression.

### 4.4 Adaptive immune dysfunction and immune evasion

CD8+ T-cell dysfunction in the tumor microenvironment spans three overlapping phenomena: phenotypic exhaustion (co-expression of PD-1, TIM-3, LAG-3, and TIGIT), functional anergy (reduced IFN-γ, granzyme B, and perforin), and physical exclusion from tumor nests. M2 TAM infiltration linked to GNA15, established so far only correlatively in ovarian cancer, is at least consistent with all three, since established TAM biology alone could explain each: IL-10 suppresses IFN-γ production by CD8+ T cells, TGF-β upregulates exhaustion markers, and TAM-driven matrix remodeling together with VEGF-driven aberrant vasculature can exclude T cells from the tumor nest. None of this, however, has been directly examined in GNA15-high tumors; CD8+ infiltration and exhaustion-marker expression have not been measured in that setting. Treg accumulation raises a similarly unexplored possibility, one that would be expected to follow CCL17/CCL22-driven recruitment and TGF-β-induced FOXP3 expression, but no published dataset has measured either variable in GNA15-stratified specimens. Because MDSC and Treg biology both operate through cytokine circuits already implicated upstream in GNA15-driven myeloid reprogramming (IL-6, TGF-β, STAT3), it is more parsimonious to treat these adaptive-arm processes as extensions of a single paracrine hypothesis than as independent mechanisms requiring separate validation. NK-cell biology illustrates this evidentiary framework with particular clarity, because two independently established immunological pathways predict opposite effects on NK cell-mediated cytotoxicity in GNA15-high tumors, and neither has been tested directly in a GNA15 model. Pathway A (EMT–MHC-I–missing-self) begins from the established principle that NK cells preferentially eliminate targets displaying reduced surface MHC class I through the well-characterized “missing-self” mechanism, since MHC-I engagement of inhibitory killer-cell immunoglobulin-like receptors and NKG2A normally restrains NK-cell cytotoxicity. In canonical EMT programs, the transcription factors Snail and ZEB1 suppress components of the antigen-presentation and MHC-I machinery. If the EMT-associated transcriptional signature already documented in the GNA15-high TNBC S2 cluster (14) were accompanied by this canonical Snail/ZEB1-mediated MHC-I downregulation, it would be expected, by extension of well-established general NK biology, to render those mesenchymal tumor cells more susceptible to NK cell-mediated cytotoxicity; GPCR signaling more broadly is already known to shape TNBC’s immunosuppressive tumor immune context through chemokine, sphingosine-1-phosphate, and prostaglandin E2 receptors (34). This chain of inference rests on two facts, each independently established in the general cancer immunology literature (EMT-associated MHC-I loss and missing-self recognition), joined by an untested link: whether GNA15-driven EMT actually produces functional MHC-I loss in any tumor type. It should accordingly be read as mechanistically plausible rather than demonstrated. Pathway B (TGF-β–activating-receptor suppression) works in the opposite direction. TGF-β is a well-characterized suppressor of NK-cell effector function, acting in part by downregulating the activating receptors NKG2D and DNAM-1 and by impairing NK-cell cytotoxic granule release and IFN-γ production. GNA15-high tumor microenvironments are hypothesized elsewhere in this review to be TGF-β-enriched, based on NF-κB/STAT3-driven cytokine output; if that hypothesis holds, the same microenvironment invoked in Pathway A to sensitize tumor cells to NK recognition would concurrently suppress the NK cells’ capacity to act on that recognition. As with Pathway A, each individual link in this chain (TGF-β abundance in GNA15-high tumors; TGF-β-mediated suppression of NKG2D/DNAM-1) is supported by evidence outside the GNA15 literature, but the combined pathway has not been demonstrated in a GNA15-specific system. Because Pathways A and B have not been measured in the same tumor, this review does not, as a general rule, adjudicate which predominates in GNA15-high disease. The available immunology literature instead suggests that the net effect is likely to be tumor-type- and context-dependent, governed by the relative balance of: (i) the degree of tumor-cell MHC-I loss actually achieved by GNA15-associated EMT, if any; (ii) the completeness of the EMT program itself, since partial EMT states may not reach the transcriptional threshold required for MHC-I suppression; (iii) local TGF-β abundance and bioavailability; (iv) the density and functional state of NK-cell infiltration, since a suppressed microenvironment may exclude NK cells before either pathway becomes relevant; (v) surface expression of NK activating receptors and their ligands on the tumor cells themselves; and (vi) the broader suppressive myeloid and stromal context, including M2 TAM density, which can act on NK cells through mechanisms independent of TGF-β. A comparable spatial antagonism between myeloid and NK compartments has been described in non-small cell lung cancer, where anti-inflammatory macrophages and NK/T cells occupy inverse spatial distributions and macrophage dominance tracks with reduced NK cell-mediated cytotoxicity (35), offering a tractable analogy, though not evidence, for GNA15-high, TAM-enriched tumors. Resolving which pathway predominates, and under which conditions, will require comparative experimental studies that measure tumor-cell MHC-I and NK-ligand expression, TGF-β levels, and NK-cell functional output side by side within the same GNA15-stratified specimens or models, rather than inferring the outcome from either pathway in isolation. The answer carries direct implications for CAR-NK and NK-engaging approach design in GNA15-high disease. Because GNA15 is not expressed in lymphocytes, any of the dysfunctions discussed above would, in any case, have to act indirectly, through tumor-intrinsic and stromal mechanisms, unlike the Gαs–PKA axis, which suppresses T-cell receptor signaling cell-intrinsically (5). Antigen presentation is a further point of vulnerability that extends beyond NK recognition. In canonical EMT models, Snail and ZEB1 suppress antigen-presentation machinery at multiple levels, and EMT-associated TGF-β further impairs both NK- and T-cell effector function (8, 14), but whether GNA15 signaling actually induces functional EMT with consequent MHC-I loss in any tumor type has not been shown, as noted above. The implications of that open question are more concrete than the underlying mechanism itself. Bispecific antibodies that redirect T cells independently of MHC-I would sidestep antigen-presentation loss but not the T-cell exclusion imposed by a macrophage-dominant, TGF-β-rich stroma, and CAR-T cells would encounter the same trafficking barriers due to EMT-associated matrix remodeling. CAR-NK cells, which resist TGF-β suppression more effectively than CD8+ T cells in other tumor contexts and are naturally suited to MHC-I-low targets, represent a more rational candidate in this scenario, though this remains a hypothesis for systematic testing rather than a settled conclusion (**Figure 2**).

**Figure 2:**
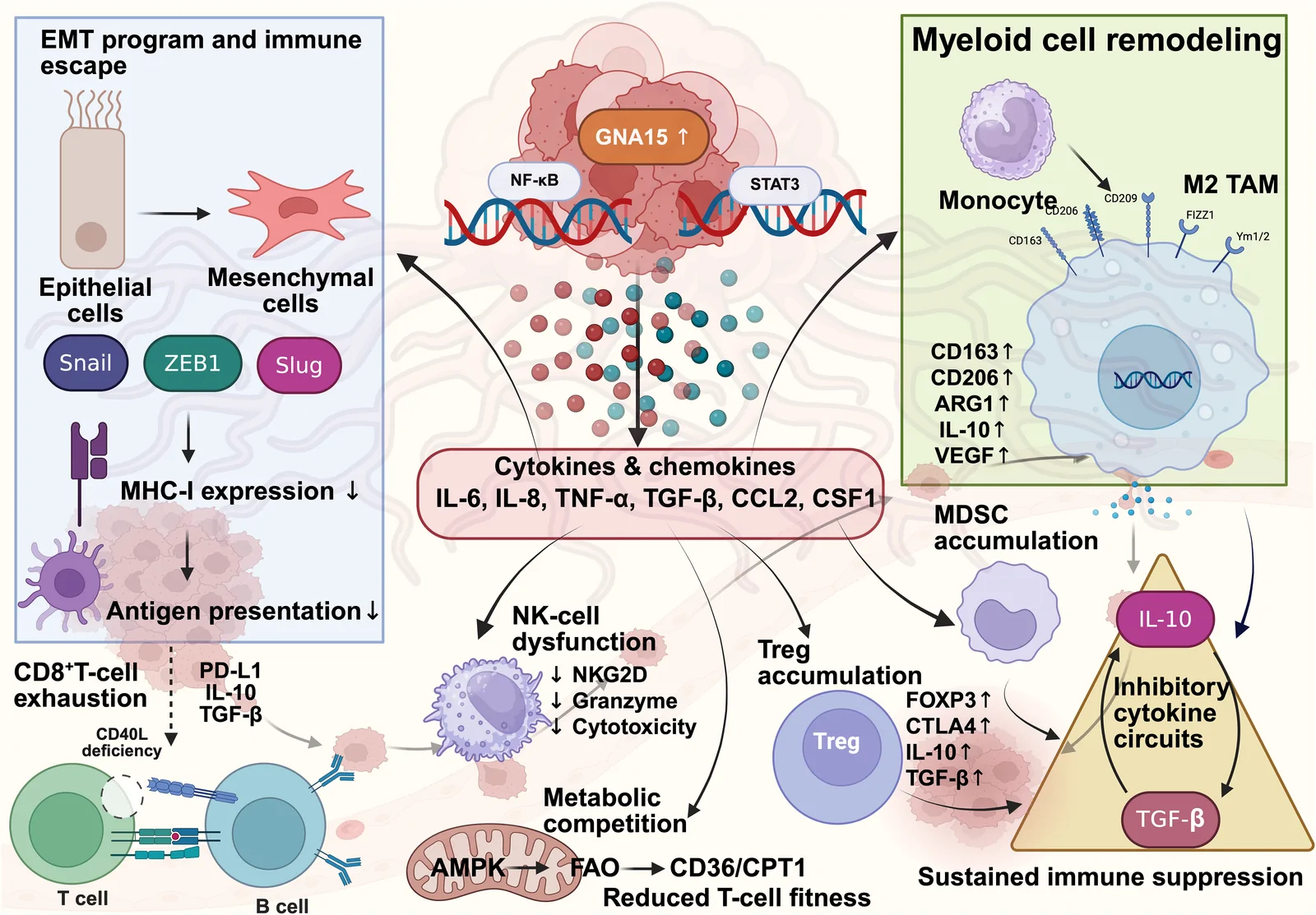
Proposed mechanisms of GNA15-mediated tumor immune microenvironment remodeling. This figure illustrates how GNA15 overexpression could build an immunosuppressive tumor microenvironment through coordinated inflammatory signaling and cellular crosstalk. NF-kappaB and STAT3 activation drive cytokine and chemokine production, including IL-6, IL-8, TNF-alpha, TGF-beta, CCL2, and CSF1, which may recruit and polarize tumor-associated macrophages toward an M2 phenotype and are compatible with the accumulation of myeloid-derived suppressor cells and regulatory T cells. Potential downstream consequences include exhaustion-driven loss of cytotoxic lymphocyte activity, suppression of natural killer cell effector function, and reinforcement of inhibitory cytokine circuits. Concurrent epithelial–mesenchymal transition (EMT) programs are associated with reduced antigen presentation and diminished MHC-I expression, providing an additional route to immune escape. These processes may contribute to the establishment of an immunosuppressive tumor microenvironment marked by immune exclusion, checkpoint activation, impaired tumor recognition, and blunted anti-tumor immunity. (Solid black arrows indicate established mechanisms, supported by direct experimental evidence (genetic silencing, pharmacological inhibition, or validated correlation) in a GNA15 model system; dashed arrows indicate hypothetical or inferred mechanisms, extrapolated from indirect evidence or from biology established in other contexts that has not yet been directly tested for GNA15; gray arrows indicate paracrine signaling mediated by soluble factors between cell types; the navy arrow indicates a tumor-associated macrophage (TAM)–derived contribution to immunosuppression; and ↑/↓ indicate increased and decreased expression or activity, respectively).

### 4.5 Checkpoint biology

No published study has directly tested GNA15’s effect on PD-L1, PD-1, CTLA-4, TIGIT, LAG-3, or TIM-3 expression through gain- or loss-of-function experiments with matched checkpoint quantification. PD-L1 transcription is governed by NF-κB (direct promoter binding), STAT3 (required for IFN-γ-independent induction), MAPK/MEK/ERK (mRNA stabilization), and PI3K/AKT/mTOR (translational enhancement) (36, 37), and GNA15 activates all four of these nodes (8, 13, 14). Integration of four independent routes to PD-L1 induction makes GNA15 a plausible, though still unconfirmed, checkpoint regulator; no systematic comparison across candidate oncogenes has established how unusual this integration actually is. Whether it translates into differential anti-PD-1/PD-L1 responsiveness depends on whether the broader tumor microenvironment remains permissive for T-cell reinvigoration after checkpoint release, a condition that GNA15-driven M2 polarization could undermine. CTLA-4 sets the amplitude of T-cell priming in secondary lymphoid organs and is expressed constitutively on Tregs and transiently on activated conventional T cells. A GNA15-driven route to Treg accumulation, inferred from hypothetical TGF-β upregulation via STAT3/NF-κB activation, would be consistent with amplified CTLA-4-mediated suppression of T-cell priming, though this remains an inference rather than a demonstrated pathway. Anti-CTLA-4 therapy (ipilimumab, tremelimumab) depletes intratumoral Tregs through FcγR-dependent mechanisms and releases that priming restraint. This raises the untested possibility that GNA15-high, Treg-enriched tumor microenvironments are contexts in which anti-CTLA-4 combined with anti-PD-1 is more effective by addressing both priming and effector checkpoint barriers. TIGIT, LAG-3, and TIM-3 are next-generation checkpoint targets whose transcriptional regulation overlaps substantially with that of PD-L1 (NF-κB, STAT3, MAPK). Their co-expression with PD-L1 on tumor cells and exhausted T cells is a feature of deeply immunosuppressive tumor microenvironments (36). GNA15-high tumors with concurrent M2 TAM enrichment and EMT activation are plausible candidates for this high-checkpoint configuration and merit evaluation for combinatorial blockade, though it remains entirely unstudied whether GNA15 actually regulates any of these secondary checkpoints. CTLA-4, PD-1, and these newer targets act at sequential stages of the T-cell response, so GNA15’s eventual clinical relevance will likely depend on identifying which stage its downstream signaling affects, rather than on any single checkpoint interaction (**Table 2**).

**Table 2:** Cancer-specific evidence supporting GNA15-mediated oncogenesis and immune regulation.

Cancer type	GNA15 mechanism	Immune/TME impact	Prognostic value	Therapeutic targets	References
AML	p38–MK2–CREB axis; LSC enrichment	NF-κB–cytokine axis; potential paracrine IL-6/IL-8 immunosuppression in BM niche	Independent OS/RFS predictor (multivariate)	p38i; MEK/ERK inhibitors; CRISPR silencing	(13)
Thyroid carcinoma	GNA15–BTK coupling; pan-MAPK (ERK/JNK/p38) activation	BTK-dependent NF-κB; hypothetical PD-L1 upregulation	Aggressive clinicopathological correlate (Tier II)	BTK inhibitors; MEK inhibitors	(12)
Ovarian cancer	M2 TAM polarization; STAT3/NF-κB activation	CD163/CD206 enrichment; immune-excluded microenvironment	OS-predictive in a programmed cell death (PCD)-related gene signature	Anti-PD-1/PD-L1 + STAT3i; macrophage repolarization	(9, 10)
TNBC	scRNA-seq malignant cluster (S2): hub gene for malignancy-related pathways	Hypothesized TME remodeling and EMT-linked MHC-I suppression; not directly assessed	Marks aggressive subpopulation	MEK inhibitors; CAR-NK (MHC-I-low context); CAR-T sensitization (hypothetical; no GNA15-specific data)	(14)
PDAC	Epigenetic promoter demethylation; lineage plasticity; invasion	Immune recognition impairment; desmoplastic immune exclusion correlates	Poor OS; disease progression	DNMT inhibitors; CRISPR; FAO inhibitors	(8)
B-ALL	AMPK to CPT1/2 to FAO; CD36 upregulation; glycerophospholipid rewiring	Metabolic competition with TME T cells (mechanistic hypothesis); CD36 as surface target	Drug resistance correlates	Etomoxir; dorsomorphin; SBI-0206965; selective CPT1 inhibitors	(15)

## 5 Therapeutic strategies stratified by evidentiary strength

### 5.1 Established rationale: genetic validation and downstream effector inhibition

shRNA or CRISPR/Cas9 silencing of GNA15 has reproducibly reversed tumorigenic phenotypes in PDAC, AML, ovarian cancer, and TNBC (8, 9, 13, 14), the most direct genetic validation of GNA15 as a therapeutic node in any tumor type reviewed here. Clinical translation faces one specific obstacle, because GNA15 is physiologically expressed in hematopoietic cells, systemic RNAi delivery would require tumor-selective nanoparticulate targeting to avoid on-target toxicity. CRISPR/Cas9 mediated editing of the GNA15 promoter may provide a more durable strategy for suppressing its expression, although efficient *in vivo* delivery remains a major challenge. Among downstream therapeutic approaches, the GNA15–AMPK–FAO pathway in B-ALL currently has strongest pharmacological support. Although etomoxir effectively reversed chemoresistance in preclinical models, its dose-limiting hepatotoxicity restricts clinical applicability, making next-generation CPT1 inhibitors or lower-dose combination regimens more practical alternatives. Likewise, the ability of dorsomorphin to counteract GNA15-driven FAO highlights the potential of more selective AMPK inhibitors (SBI-0206965) (15, 38). Nevertheless, the context-dependent and, in some settings, tumor-suppressive functions of AMPK warrant careful evaluation before clinical translation (39). Existing MAPK-targeted agents, including MEK inhibitors (trametinib, cobimetinib, and binimetinib), ERK inhibitors (ulixertinib, and RAF inhibitors), provide clinically established options for targeting GNA15-driven MAPK signaling. However, because ERK, JNK, and p38 are activated simultaneously in thyroid carcinoma, inhibition of a single downstream kinase is unlikely to provide sustained benefit owing to compensatory pathway activation. A more rational approach would involve concurrent inhibition of multiple MAPK branches or upstream intervention at the GNA15–BTK signaling axis before downstream pathway divergence. Given the experimentally validated interaction between GNA15 and BTK in thyroid carcinoma, BTK inhibitors represent a promising therapeutic strategy for GNA15-high solid tumors (12, 40). In parallel, biomarker-driven basket trials using GNA15 expression as a patient-selection criterion could assess this strategy across diverse malignancies. In PDAC, promoter hypomethylation contributes to GNA15 upregulation (8), suggesting that clinically available DNMT inhibitors, including azacitidine and decitabine, might restore promoter methylation and attenuate GNA15-dependent oncogenic signaling. However, whether such epigenetic intervention would also alleviate immune evasion cannot be determined until a causal role for GNA15 in immune regulation has been established.

### 5.2 Checkpoint blockade and combination strategies

Immune checkpoint inhibitors achieve their greatest benefit in tumors enriched for activated T cells and reduced efficacy in macrophage-dominant, immunosuppressive microenvironments. This immune landscape resembles that reported in GNA15-high ovarian cancer (9). Activation of NF-κB and STAT3 downstream of GNA15 provides a plausible mechanistic basis for the elevated PD-L1 expression observed in GNA15-high tumors (36, 37), although direct experimental validation is still lacking. Because PD-1/PD-L1 inhibitors are already approved for ovarian cancer, TNBC, and AML, prospective clinical studies incorporating GNA15 expression, PD-L1 quantification, immune-cell deconvolution, and treatment outcomes would provide the most direct assessment of whether GNA15 predicts responsiveness to checkpoint blockade. A similarly compelling rationale exists for combining checkpoint inhibitors with STAT3-targeted therapies. If GNA15-dependent IL-6–STAT3 signaling simultaneously sustains PD-L1 expression and promotes M2 macrophage polarization, then STAT3 inhibition could reduce immunosuppressive cytokine production, repolarize tumor-associated macrophages toward an M1 phenotype, and thereby enhance the efficacy of anti-PD-1 immunotherapy. That is still a hypothesis, and testing it will require immune-competent preclinical models. Anti-CTLA-4 paired with anti-PD-1 in GNA15-high, Treg-enriched tumors is a similarly reasonable idea that is, just as much, untested. Cholesterol and lipid metabolism, which intersect AMPK/FAO biology, add a further avenue worth investigating, excess cholesterol in tumor-associated macrophages drives M2 reprogramming and CD8+ exhaustion through XBP1 in other tumor contexts (33), a pathway that converges on the same GNA15-associated metabolic axis described above, though this integration has not been demonstrated specifically in GNA15-high disease.

### 5.3 Emerging approaches: cellular and antibody-based approaches with partial support

Bispecific antibodies that simultaneously engage tumor-surface antigens and T-cell CD3 or NK-cell CD16 redirect immune effectors to tumor cells independently of MHC class I presentation, a property that may be particularly relevant where GNA15-associated EMT could limit TCR-dependent cytotoxicity (14). Bispecific T-cell engager (BiTE) therapy, exemplified by blinatumomab in B-cell malignancies and the DLL3-targeting agent tarlatamab in small cell lung cancer (41), demonstrates that MHC-independent tumor recognition can produce clinically meaningful responses in other settings. BiTE efficacy, however, is substantially impaired by immune checkpoint upregulation and sparse T-cell infiltrates in immunosuppressive tumor microenvironments, both of which would plausibly apply to GNA15-high tumors given their immunosuppressive features (42). GNA15 itself, as an intracellular Gα subunit, is not a druggable bispecific target, but GNA15-associated surface phenotypes may reveal one. Elevated CD36 expression in B-ALL (15) and the distinct surface marker profile of the GNA15-high TNBC S2 cluster (14) are both plausible leads, with CD36 the more immediately actionable candidate given its existing metabolomic validation (15); functional confirmation as a bispecific target, rather than simply a correlate, would still be required. Among cell-based immunotherapies, CAR-NK and CAR-macrophage (CAR-M) platforms currently present the strongest mechanistic rationale for GNA15-high malignancies, supported by the evidence discussed throughout this review rather than by extrapolation from unrelated tumor models (43). CAR-M therapies, designed either to promote tumor cell phagocytosis or to remodel an immunosuppressive tumor microenvironment, are particularly relevant given the predominance of M2-like tumor-associated macrophages reported in GNA15-high ovarian cancer (9). Conceptually, simultaneous tumor clearance and macrophage repolarization toward an M1 phenotype may create a microenvironment more permissive to subsequent immunotherapies, including immune checkpoint blockade or CAR-T-cell treatment, although this therapeutic strategy remains to be evaluated experimentally. If certain tumor subpopulations with high GNA15 expression, activated EMT status, and low MHC-I levels are confirmed, patients with these tumors might benefit more from CAR-NK or NK-engaging bispecific antibodies rather than CAR-T or standard checkpoint inhibitors. However, this remains a hypothesis that needs direct experimental validation.

### 5.4 Hypothesis-generating concepts

Antibody-drug conjugates (ADCs) rely on tumor-specific surface antigen expression, and an increasing number are approved, with additional ones in development (44). While GNA15’s intracellular localization suggests it is not a direct target, the surface antigen profile of GNA15-high malignant clusters identified through scRNA-seq (14) could include potential candidates for future research. A major concern is cross-resistance, in which GNA15-driven FAO-mediated survival signaling (15) suggests that tumors with high GNA15 levels might resist ADC payloads that act via mechanisms similar to traditional DNA-damaging chemotherapy. Although this hypothesis stems from the metabolic biology discussed earlier, it has not yet been empirically tested. ADCs can also engage innate immunity through Fc-mediated mechanisms and immunogenic cell death that primes adaptive responses; the anti-apoptotic signaling downstream of CREB (BCL2, MCL1) (13) that characterizes GNA15-high tumors may blunt this secondary immune priming effect, a question that has not yet been assessed but is directly testable. TCR-engineered T cells, including conventional CAR-T cells, are likely to encounter difficulties when GNA15-driven EMT results in MHC-I loss. Since antigen-specific recognition depends on presentation machinery, which these mesenchymal subpopulations might lack, ongoing therapy could inadvertently select for clones that evade immune detection. This remains a hypothesis and not a confirmed limitation. Using unconventional effectors could provide an alternative approach. γδ T cells, NKT cells, and innate lymphoid cells (ILCs) recognize non-peptide or stress-associated antigens through mechanisms that do not depend on MHC, so they might stay active where conventional CD8+ T cells fail, sidestepping GNA15-driven loss of antigen presentation and resisting some of the immunosuppressive cytokines in play. Their utility in GNA15-high disease specifically has not been explored, and in the absence of GNA15-specific mechanistic grounding, they represent a lower near-term investigative priority than the CAR-NK and CAR-M concepts described above (**Tables 3**, **4**).

**Table 3:** Evidence appraisal approach applied to GNA15 biology.

Evidence category	Examples from GNA15 biology
Established evidence	GNA15 to p38 to MK2 to CREB axis in AML (functional rescue confirmed) (13); GNA15 to AMPK to FAO to chemoresistance in B-ALL (pharmacological validation with etomoxir) (15); GNA15–BTK functional coupling in thyroid carcinoma (rescue experiments) (12); M2 TAM infiltration correlation with GNA15 in ovarian cancer (IHC/transcriptomics) (9).
Emerging evidence	GNA15-high TNBC malignant cluster (S2) associated with malignancy-related pathways and validated by knockdown experiments (single-cell multiomics) (14); GNA15 prognostic value in the PCD gene signature for ovarian cancer (10); epigenetic promoter demethylation driving GNA15 expression in PDAC (8); EMT and MHC-I status of the GNA15-high TNBC cluster not directly assessed in the source data and remains a hypothesis.
Mechanistic hypotheses	GNA15 to NF-κB/STAT3 to PD-L1 upregulation (no direct evidence in any GNA15 model system); GNA15-driven FAO creating metabolic T-cell exclusion (biologically plausible, unvalidated); BTK-independent NF-κB sustaining PD-L1 under ibrutinib therapy (untested); Treg accumulation via TGF-β upregulation downstream of GNA15 (a three-step inference chain with no direct data at any link).
Knowledge gaps	Absence of immune-competent *in vivo* GNA15 models; no direct GNA15–PD-L1 mechanistic data; undefined paracrine M2-polarizing signals from GNA15-high tumor cells; incompletely characterized GNA15 interactome; no standardized clinical biomarker quantification approach; MDSC and Treg infiltration in GNA15-stratified tumors unassessed.

**Table 4:** GNA15 biology and implications for targeted effector approaches, stratified by evidentiary strength.

Approach	Evidence tier (Section 5)	Biological rationale	Opportunities	Limitations/evidence gaps
Downstream effector inhibitors (BTK, MEK/ERK, AMPK/CPT1)	Established rationale (5.1)	Direct genetic/pharmacological validation in GNA15 models: BTK–MAPK coupling in thyroid carcinoma (12); AMPK–FAO axis in B-ALL (15); MAPK output across tumor types (8, 13, 14)	Agents already in clinical use; biomarker-stratified basket trials feasible now	Compensatory pathway reactivation likely with single-node inhibition; immunological consequences of these agents in GNA15-high tumors untested
ICI (anti-PD-1/L1, anti-CTLA-4) + STAT3i combinations	Strong mechanistic rationale (5.2)	GNA15-driven NF-κB/STAT3 may upregulate PD-L1 (inference); M2 TAM-enriched TME in GNA15-high ovarian cancer (9)	GNA15 as a candidate, untested predictive biomarker for ICI eligibility; STAT3i to reverse M2 polarization; anti-CTLA-4 for Treg-enriched contexts	Undemonstrated; M2-dominant TME may limit T-cell reinvigoration; no clinical immunotherapy cohort data
Bispecific antibodies (BiTEs)	Emerging approach (5.3)	MHC-I-independent tumor recognition circumvents EMT-associated antigen presentation loss in GNA15-high cells	Surface proteomics of GNA15-high scRNA-seq clusters to identify novel BiTE antigens; CD36 as candidate surface target in B-ALL (15)	GNA15 is intracellular and not directly targetable; GNA15-high surface antigen inventory undefined; immunosuppressive TME reduces T-cell availability (42)
CAR-NK cells	Emerging approach (5.3)	NK cells resist TGF-β suppression better than CD8+ T cells; activation is linked to MHC-I downregulation associated with GNA15-driven EMT (14)	Preferred in GNA15-high, MHC-I-low tumor states; off-the-shelf feasibility (45, 46)	No GNA15-specific CAR-NK data; GNA15’s effect on NK ligand expression is uncharacterized
CAR-M/macrophage-based	Emerging approach (5.3)	GNA15-high, M2-enriched TME in ovarian cancer (9) is a rational context for macrophage repolarization therapy	Could eliminate tumor cells and repolarize the TAM immune context, combined with checkpoint blockade	CAR-M technology is in early clinical development; GNA15-specific CAR-M studies are absent
ADCs	Hypothesis-generating (5.4)	GNA15-associated surface antigens on malignant clusters (scRNA-seq) may be ADC-targetable	Surface proteomics to prioritize ADC targets; immunogenic cell death potential to prime adaptive immunity	GNA15 FAO-driven chemoresistance may confer cross-resistance to cytotoxic payloads (44); BCL2/MCL1 upregulation may suppress ICD
Conventional CAR-T/TCR-T	Hypothesis-generating (5.4)	GNA15-associated M2 TAM infiltration (9) and hypothesized EMT/MHC-I loss may impair persistence and antigen recognition	GNA15 pathway inhibition (MEK, STAT3, FAO) as a sensitizing co-treatment	No co-culture or *in vivo* CAR-T experiments in GNA15-high models; antigen escape and trafficking barriers unevaluated

## 6 Intratumoral heterogeneity and biomarker translation

Single-cell transcriptomics has established that intratumoral cancer-cell populations comprise distinct cell states with distinct proliferative, invasive, metabolic, and immune-interactive properties (14). The GNA15-high TNBC malignant cell cluster (S2), associated with multiple malignancy-related pathways and validated by knockdown experiments, demonstrates that GNA15 overexpression marks one such specific cellular state rather than generalized transcriptional heterogeneity (14). Whether analogous GNA15-defined cell states exist within the tumor hierarchies of ovarian cancer, AML, and PDAC cannot be determined from the currently available data. It is similarly unresolved whether the S2 cluster itself carries the EMT and MHC-I-low phenotype proposed elsewhere in this review. Resolving this distinction has concrete stakes: confirming a mesenchymal, MHC-I-low state would favor NK-dependent approaches, including CAR-NK cells and NK-engaging bispecific antibodies, over conventional T-cell-dependent approaches such as checkpoint inhibitors, CAR-T, and BiTEs. It would also clarify whether the immunosuppressive associations already documented for GNA15 in ovarian cancer and AML reflect the same underlying cellular program identified in the TNBC S2 cluster, or distinct tumor-specific manifestations of a shared signaling node. Spatial transcriptomics is the method best suited to determining how GNA15-expressing tumor states relate to adjacent immune infiltrate composition and to the exclusion patterns that ultimately determine which effector approaches can reach the tumor, connecting the correlative findings described above to tissue-level ground truth (**Table 5**). Clinical adoption of GNA15 as a biomarker faces a practical obstacle rather than a biological one. Its independent prognostic value across AML, PDAC, thyroid carcinoma, ovarian cancer, and TNBC supports its use as a marker of a recurrent oncogenic cellular state. This prognostic role should be kept conceptually distinct from predictive biomarker status: a predictive biomarker forecasts differential benefit from a specific therapy, and no dataset linking GNA15 expression to treatment response currently exists for any therapeutic modality discussed in this review. The evidence base behind GNA15’s prognostic associations itself consists of small, retrospective, and heterogeneous cohorts spanning different tumor types, non-standardized immunohistochemistry, qRT-PCR, and transcriptomic quantification methods, the absence of a harmonized positivity cut-off, and, to date, no prospective validation cohort. Moving GNA15 from prognostic correlate to either a validated prognostic tool or a predictive biomarker will require standardized quantification, cross-cohort and prospective validation, head-to-head comparison against established prognostic markers in each tumor type, and, specifically for predictive use, cohorts with matched treatment and outcome data. Companion diagnostic development should proceed alongside the therapeutic targeting strategies described elsewhere in this review, so that GNA15-stratified basket trials, particularly for immuno-oncology combinations where GNA15 might help define microenvironmental composition and inform checkpoint-trial eligibility, become feasible once this validation work, and the underlying causal biology, are further along. Single-cell multiomics profiling, already applied to GNA15-stratified pediatric leukemia cohorts (47), offers a template that other tumor types could adopt directly (**Figure 3**).

**Table 5:** Knowledge gaps and future research priorities.

Knowledge gap	Experimental approach	Translational relevance	Approach implication
No immune-competent *in vivo* GNA15 models	Syngeneic orthotopic GNA15 overexpression; conditional knock-in; humanized PDX	Required to evaluate TME remodeling and immunotherapy combination efficacy in a physiological context	Checkpoint inhibitors; CAR-T/NK combination design
GNA15–PD-L1 regulatory axis unexplored	GNA15 gain/loss-of-function + PD-L1 quantification (flow/IHC); CUT&RUN at the PD-L1 promoter for NF-κB/STAT3 binding	Determines ICI eligibility prediction in GNA15-high disease	Anti-PD-1/L1; predictive biomarker development
Mechanism of GNA15-driven M2 polarization undefined	Conditioned-medium transfer; cytokine array; scRNA-seq of co-culture TAMs stratified by GNA15	Identifies paracrine immunosuppressive mediators for neutralization	Macrophage repolarization agents; CAR-T sensitization
MDSC and Treg infiltration in GNA15-high tumors not assessed	Immune deconvolution of existing GNA15-stratified transcriptomic datasets; flow cytometry of GNA15-stratified clinical specimens	Defines the full scope of GNA15-driven immune suppression; informs anti-CTLA-4 combination eligibility	Anti-CTLA-4; combination immunotherapy design
Metabolic competition between GNA15-high tumor cells and T cells not assessed	Seahorse metabolic flux assays in co-cultures; FAO inhibition with T-cell functional rescue experiments	FAO inhibition as an immune-sensitizing strategy	CAR-T/NK combination therapy; metabolic checkpoint targeting
GNA15 surface antigen correlates on malignant clusters not identified	Surface proteomics (mass spectrometry) of GNA15-high vs. GNA15-low populations from scRNA-seq	ADC target identification on GNA15-high subpopulations	Bispecific antibodies; ADC design
NK ligand expression on GNA15-high cells uncharacterized	NKG2D/DNAM-1 ligand surface expression profiling in GNA15-stratified tumor cells; NK cell-mediated cytotoxicity assays	Determines CAR-NK vs. CAR-T approach selection in GNA15-high, MHC-I-low disease	CAR-NK therapy; NK-engaging bispecific antibodies
No standardized clinical GNA15 biomarker approach	Harmonized IHC scoring; qRT-PCR validation across TCGA, ICGC, and prospective cohorts	Companion diagnostic development; GNA15-stratified basket trial design	All targeted immunotherapy approaches

**Figure 3:**
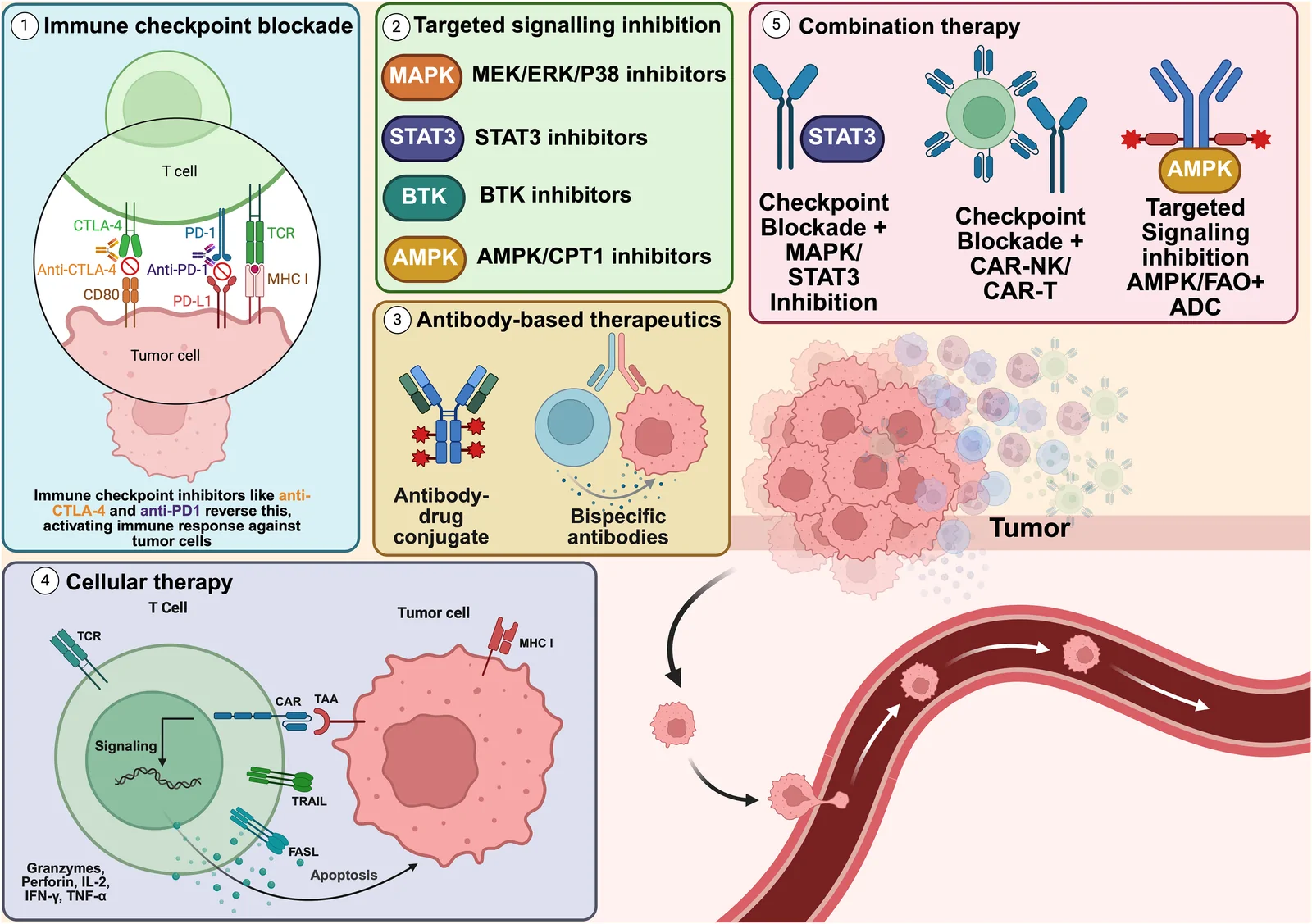
Therapeutic framework for GNA15-guided biomarker development and translational stratification. An increase in GNA15 expression marks an aggressive biological state characterized by immunosuppression, metabolic plasticity, EMT activation, and resistance-associated signaling. Together, these tumor-intrinsic and microenvironmental features may inform treatment selection among targeted immunotherapeutic strategies, including immune checkpoint inhibitors, CAR-T cells, CAR-NK cells, bispecific antibodies, antibody–drug conjugates, and macrophage-based cellular therapies. It provides a framework for understanding how GNA15-associated tumor states may influence therapeutic responsiveness and highlights rational combination strategies for future investigation, including immune checkpoint blockade with STAT3 inhibition, macrophage repolarization, and pharmacological targeting of the MAPK, BTK, AMPK, or CPT1 signaling pathways. Beyond its therapeutic relevance, GNA15 may serve as a candidate biomarker for patient stratification, prognostic assessment, treatment selection, and response monitoring, illustrating how mechanistic insights into GNA15 biology could support precision immuno-oncology. (⊘ indicates blockade of a receptor–ligand interaction by an immune checkpoint inhibitor; * indicates a cytotoxic payload conjugated to an antibody; Y-shaped icons represent antibodies (monoclonal, bispecific, or antibody–drug conjugate, as indicated); black arrows indicate the direction of a cellular or therapeutic process; and white arrows indicate the direction of cell trafficking within the vasculature).

## 7 Limitations and future priorities

GNA15’s multi-effector signaling organization cuts both ways for the therapeutic strategies described above. The same simultaneous engagement of MAPK, FAO, and BTK-dependent signaling that opens multiple entry points also means single-pathway suppression may be insufficient to shut down GNA15-driven oncogenic and immunosuppressive programs; compensatory pathway reactivation is a predictable resistance mechanism (12). GNA15’s physiological expression in hematopoietic cells poses a genuine on-target toxicity risk for any systemic targeting strategy, which is why tumor-selective delivery or downstream effector targeting is generally preferable to directly inhibiting Gα15. No selective small-molecule inhibitor targeting Gα15 has been developed to date, and its discovery remains a major challenge in chemical biology. Furthermore, with the exception of a single AML xenograft model, none of the malignancies discussed in this review has been investigated using immune-competent *in vivo* systems. Consequently, all proposed links between GNA15 and tumor immunity are based on correlative clinical observations and mechanistic inference from established signaling networks rather than direct evidence of causality. This limitation is compounded by the fact that most available studies rely on small, retrospective, and non-standardized patient cohorts, leaving the current evidence base largely exploratory rather than definitive. Moreover, no investigation has simultaneously examined GNA15 expression alongside tumor-infiltrating lymphocyte abundance and composition, immune checkpoint expression, tumor-associated macrophage polarization, and clinical responses to immunotherapy within a single cohort. These gaps substantially limit our understanding of whether GNA15 influences the efficacy of targeted effector therapies. Addressing these questions will require developing immune-competent experimental models, particularly syngeneic orthotopic tumors and humanized PDX systems, which is a high priority for future studies. These models are the only practical way to directly study GNA15-driven changes in the tumor microenvironment, checkpoint control, and the effectiveness of combination immunotherapies under physiologically relevant conditions, rather than relying solely on correlative data. Within such models, pairing GNA15 gain- and loss-of-function with CUT&RUN or ChIP-seq mapping of NF-κB/STAT3 occupancy at checkpoint loci would provide the most direct test yet of whether GNA15 actually drives checkpoint expression. Three further gaps remain unresolved. Spatial transcriptomic and single-cell integration in GNA15-stratified specimens, building on the template already established in pediatric ALL (47), would clarify the spatial organization between GNA15-expressing tumor cells and immune infiltrate at tissue scale. Characterizing the GNA15 interactome by proximity labeling and phosphoproteomics would identify upstream GPCR dependencies and candidate targets, complemented by surface proteomics of GNA15-high populations to define bispecific and ADC antigens. Structural characterization of Gα15–GPCR–effector complexes by cryo-EM, addressing the noted current absence of direct structural data, could extend the chemical-probe precedent already established for Gαq (YM-254890, FR900359) (1, 19). Prospective multicenter biomarker validation, using standardized quantification and co-registered immunotherapy response data, remains the necessary final step for clinical utility, but depends on this mechanistic work being completed first. The three principal effector axes identified here, MAPK, FAO/AMPK, and BTK, are each individually druggable with agents already approved for clinical use. The practical barrier to testing the therapeutic strategies proposed in this review is therefore patient identification rather than drug availability, a gap that biomarker development would directly address. Longitudinal immune monitoring of GNA15-stratified patients receiving immunotherapy, with serial biopsies analyzed by spatial transcriptomics and single-cell sequencing, would generate the real-world data needed to test GNA15 as a candidate predictive biomarker and to identify resistance mechanisms as they emerge (48).

## 8 Conclusion

GNA15 has emerged from the field of hematopoietic signaling biology as a multi-pathway oncogenic transducer whose activities lie at the interface of tumor-intrinsic signaling and microenvironmental remodeling. Bringing together the evidence reviewed above, three levels of confidence can be distinguished. What is established. GNA15 is supported as an oncogenic signaling component by direct genetic and, in one axis, pharmacological validation across multiple malignancies. Its oncogenic output converges on four recurrent programs rather than six disease-specific mechanisms: MAPK pathway amplification, most completely characterized through the p38–MK2–CREB cascade in AML; BTK-coupled signaling, established by rescue experiments in thyroid carcinoma; AMPK-orchestrated metabolic adaptation, established genetically and pharmacologically in B-ALL; and EMT-associated cellular plasticity, identified in the TNBC S2 malignant cluster. shRNA or CRISPR/Cas9 silencing of GNA15 reproducibly reverses tumorigenic phenotypes in PDAC, AML, ovarian cancer, and TNBC, and downstream effectors of these programs (CPT1, AMPK, BTK, MEK) are now pharmacologically accessible, without waiting for a Gα15-selective inhibitor that does not yet exist. GNA15’s structural divergence from Gαq and Gα11, concentrated in the α5 helix and α4–β6 loop, provides a defensible, literature-supported basis for its distinct receptor-coupling behavior and its distinct therapeutic-targeting problem. This body of evidence is sufficient to support continued therapeutic development of GNA15 and its established downstream effectors. What is associated. Across AML, PDAC, thyroid carcinoma, ovarian cancer, TNBC, and B-ALL, GNA15 expression correlates with adverse clinical outcomes; in ovarian cancer, it further correlates with M2 macrophage infiltration and immunosuppressive transcriptional signatures. These associations are consistent and reproducible within their respective cohorts, thereby justifying GNA15’s development as a prognostic biomarker. They do not, on their own, establish causality, and the cohorts underlying them are predominantly small, retrospective, and non-standardized in GNA15 quantification. What remains unknown. Whether GNA15 directly controls tumor–immune crosstalk, determines immune-cell composition, regulates checkpoint expression, or predicts response to immunotherapy remains incompletely established. With the exception of a single AML xenograft model, none of the malignancies discussed here has been examined in an immune-competent *in vivo* system, and no study has performed GNA15 gain- or loss-of-function with matched immune readouts. The proposed links between GNA15 signaling and M2 polarization, MDSC and Treg accumulation, NK-cell susceptibility, and checkpoint regulation are mechanistically plausible extensions of pathways, NF-κB, STAT3, TGF-β, that GNA15 is independently known to activate, but none has been demonstrated directly, and this review has deliberately avoided treating that plausibility as evidence of a demonstrated mechanism. Confirming whether GNA15-high, EMT-activated, MHC-I-low tumor subpopulations show differential susceptibility to NK- versus T-cell-dependent approaches would be the single finding most likely to convert GNA15 from a prognostic correlate into a biomarker for effector-approach selection. Immune-competent *in vivo* modeling and direct interrogation of the GNA15–checkpoint axis are accordingly the most important open questions raised in this review; interactome characterization, structural analysis of GNA15–GPCR–effector complexes, spatial validation, and multicenter biomarker studies follow from that foundational work. Within the Research Topic of Targeted Effector Approaches in Tumor Immunity, GNA15 occupies an instructive position: a tumor-intrinsic oncogenic program with substantial direct evidence for its cancer-cell-autonomous functions, and a biologically plausible but still unproven candidate regulator of the tumor immune microenvironment. The integration of GPCR biology, metabolic oncology, and tumor immunology in GNA15 research makes it a mechanistically tractable subject for continued investigation, provided that its established oncogenic biology and its hypothesis-generating immunology continue to be reported and evaluated as the two distinct evidentiary categories they are.
